# Older Adults' Physical Activity and the Relevance of Distances to Neighborhood Destinations and Barriers to Outdoor Mobility

**DOI:** 10.3389/fpubh.2020.00335

**Published:** 2020-08-07

**Authors:** Erja Portegijs, Kirsi E. Keskinen, Johanna Eronen, Milla Saajanaho, Merja Rantakokko, Taina Rantanen

**Affiliations:** ^1^Faculty of Sport and Health Sciences and Gerontology Research Center, University of Jyvaskyla, Jyväskylä, Finland; ^2^School of Health and Social Studies, JAMK University of Applied Sciences, Jyväskylä, Finland

**Keywords:** mobility limitation, physical exercise, built environment, aging, walking, active aging, age-friendly community

## Abstract

**Aim:** To determine the relevance of features located close to home and further away, our aim was to study associations between older adults' physical activity and self-reported neighborhood destinations and barriers to outdoor mobility categorized by presence and maximal distance from home.

**Methods:** Cross-sectional analyses comprising men and women 79–94 years old (57%) living independently in Central Finland (*n* = 185). Self-reported physical activity was categorized into lower (≤3 h moderate activity a week) and higher (≥4 h moderate or intense activity a week) activity. Assisted by interviewers, participants located on an interactive map destinations perceived to facilitate and barriers perceived to hinder outdoor mobility in their neighborhood. Participants' home addresses were geolocated. Euclidean distances between home and reported locations were computed, and the maximal distance from home to neighborhood destinations and barriers, respectively, was categorized based using four common buffer distances, i.e., 250 m, 500 m, 750 m, and 1 km. Participants reporting destinations or barriers within and beyond the respective distance were compared with those reporting none.

**Results:** About 80% of participants reported neighborhood destinations and 55% neighborhood barriers to outdoor mobility. Barriers were generally located closer to home than destinations [median 166 m (range 25 m−6.10 km) vs. 492 m (5 m−2.7 km)]. Logistic regression analyses adjusted for age, sex, and physical performance showed that neighborhood destinations increased the odds for higher physical activity when located beyond 500 m from home [OR 2.95, 95% confidence interval (CI) 1.02–8.54], but not when located solely within 500 m (OR 1.70, 95% CI 0.30–9.61), in comparison with when reporting no destinations. In contrast, neighborhood barriers decreased the odds for higher physical activity when solely located within 500 m (OR 0.31, 95% CI 0.14–0.72), but not when any barrier was located beyond 500 m (OR 0.96, 95% CI 0.23–3.99), compared with when reporting no barriers. Associations were similar for 250-m buffer distances, but not robust for 750-m and 1,000-m buffers because of lower prevalence.

**Conclusion:** Neighborhood barriers to outdoor mobility located close to home were associated with lower physical activity of older adults, whereas barriers further away were not. Attractive destinations for outdoor mobility located further away from home correlated with higher physical activity, potentially by motivating one to go out and be physically active. Temporal relationships warrant further study.

## Introduction

With the globally aging population, healthy, and active aging is an important policy goal endorsed by WHO and the European Union (1, 2). Physical activity is an essential aspect of active aging through its role in maintaining health and function into high age (3), and also because it constitutes a vital element of many social, communal, and even cognitive activities (4). Physical activity is defined as any bodily movement actuated by skeletal muscle and requiring energy expenditure. With age, the amount of physical exercise typically declines, whereas lighter physical activities such as walking for transport or recreation become more popular (5). Providing suitable circumstances for older adults' physical activities is important because the health benefits of even light intensity activities and activity breaks in periods of inactivity are acknowledged in current physical activity guidelines (3).

According to the socioecological model of aging (6), declines in physical and cognitive capacity make older adults more vulnerable to barriers in the physical environment, and thus, may lead to lower physical activity levels (7, 8). Walking typically starts from the home and has a limited range, hence also policies of age-friendly environments and communities acknowledge the link between the immediate neighborhood environment and physical activity. Design of age-friendly environments has been advocated by organizations such as WHO and the European Union (9). They are frequently interpreted as barrier-free environments, but clear guidelines and measures for implementation of age-friendly features are not available (10, 11). Qualitative studies especially show that features such as poor walkway quality and inadequate lighting may encumber older adults' mobility (12). Conversely, an attractive environment may positively affect older adults' out-of-home mobility, for example, by providing incentives to go out (13–15). Reporting interesting destinations in the neighborhood, such as shops and parks or green areas, is associated with higher levels of physical activity (16), and reporting multiple environmental facilitators for outdoor mobility may even protect against the development of walking difficulty years later (17, 18).

Overall, the associations between environmental factors hindering or facilitating outdoor mobility and physical activity have been demonstrated, especially in the immediate home neighborhood (12, 16). However, the relevance of the geographical areas representing the neighborhood in research has been questioned (19). Moreover, definitions of access to destinations and their operationalization, prevalence, and correlates vary hugely in different studies (20). For example, presence of certain environmental features may affect outdoor mobility of older adults differently depending on whether they are self-reported or assessed more objectively using geographical resources or environmental audits (21, 22). This is because individuals often report features that are meaningful to them and do not mention personally irrelevant features, whereas more objective methods do not distinguish these.

Self-reports and more objective measures of environment complement each other. For example, self-reported long distance to services has been reported as a barrier to outdoor mobility (20) and as a predictor of long-term detrimental changes in outdoor walking ability and frequency of walking (23, 24). However, without actual spatial references, such self-reports are difficult to translate to concrete distances and may lead to misinterpretations. Studies on active means of transportation, i.e., walking and cycling, employing GPS trackers, and map-based questionnaires have shown that older adults visit services beyond common operationalization of neighborhood, i.e., 500 m and 1 km distance from home (25, 26). Therefore, it is possible that attractive environmental destinations, also when located further away, may contribute to an individual's total physical activity.

To our knowledge, few studies have considered spatial locations of participant-reported neighborhood destinations and barriers to outdoor mobility relative to the homes. Therefore, the aim of this paper is to study associations between older adults' physical activity and self-reported neighborhood destinations and neighborhood barriers to outdoor mobility categorized according to their presence and maximal distance from home. We determined whether neighborhood destinations and barriers close to home, that is, within commonly used buffer distances of 250 m, 500 m, 750 m, and 1 km, are of equal importance as those located further away, in comparison with reporting no neighborhood destinations or barriers, respectively.

## Materials and Methods

### Study Design and Participants

We report cross-sectional analyses of the Mobility and Active Aging (MIIA) study comprising older adults aged 79–93 years living independently in Jyväskylä and Muurame municipality in Central Finland (27). Data were collected by computer-assisted face-to-face home interviews in spring 2016. Participants were part of a randomly selected sample (*N* = 298) of the population-based “Life-space mobility in old age” (LISPE) cohort, which was composed 4 years earlier (28). Of those invited, 15 were not reached and 77 declined to participate. Those living independently in the recruitment area, willing to participate, and able to communicate were eligible for participation. Compared with non-participants (*n* = 642) from the original LISPE cohort, MIIA participants (*n* = 206) did not differ in terms of sex, number of chronic conditions, or years of education, but they were somewhat younger, and had slightly better cognition and physical performance than the others as reported earlier (27). Participants' home addresses were derived from the national population register and geocoded in the Geographic Information System (GIS) (29) [Digiroad dataset 2013 (30)] using ArcMap 10.3.1 (Esri, Redlands, CA, USA). This study was carried out in accordance with Finnish National Board on Research Integrity guidelines and recommendations of the European Union. The MIIA study protocol was approved by The Ethical Committee of the University of Jyväskylä. All participants gave written informed consent before the assessments in accordance with the Declaration of Helsinki.

### Main Variables

Level of *habitual physical activity* was self-reported using a validated seven-category question combining frequency and intensity of common physical activities (31). The question takes into account physical exercise as well as physical activity related to transport and household activities. Participants were asked to choose the description that best captured their level of physical activity in the previous 6 months. Response options were (0) mostly resting, hardly any activity, (1) mostly sitting, (2) light physical activity, (3) moderate physical activity about 3 h a week, (4) moderate physical activity at least 4 h a week or heavier physical activity up to 2 h a week, (5) Engaging in active sports several times a week making you sweat and breathless or doing heavy gardening or leisure-time activities (at least 3 h a week), and (6) Practicing competitive sports. For category 1 to 4, additional examples of eligible activities were provided. In line with earlier studies, participants were categorized into lower (≤3 h moderate activity a week; category 0–3) and higher (≥4 h moderate or intensive activity a week; category 4–6) (31).

**Figure 1:**
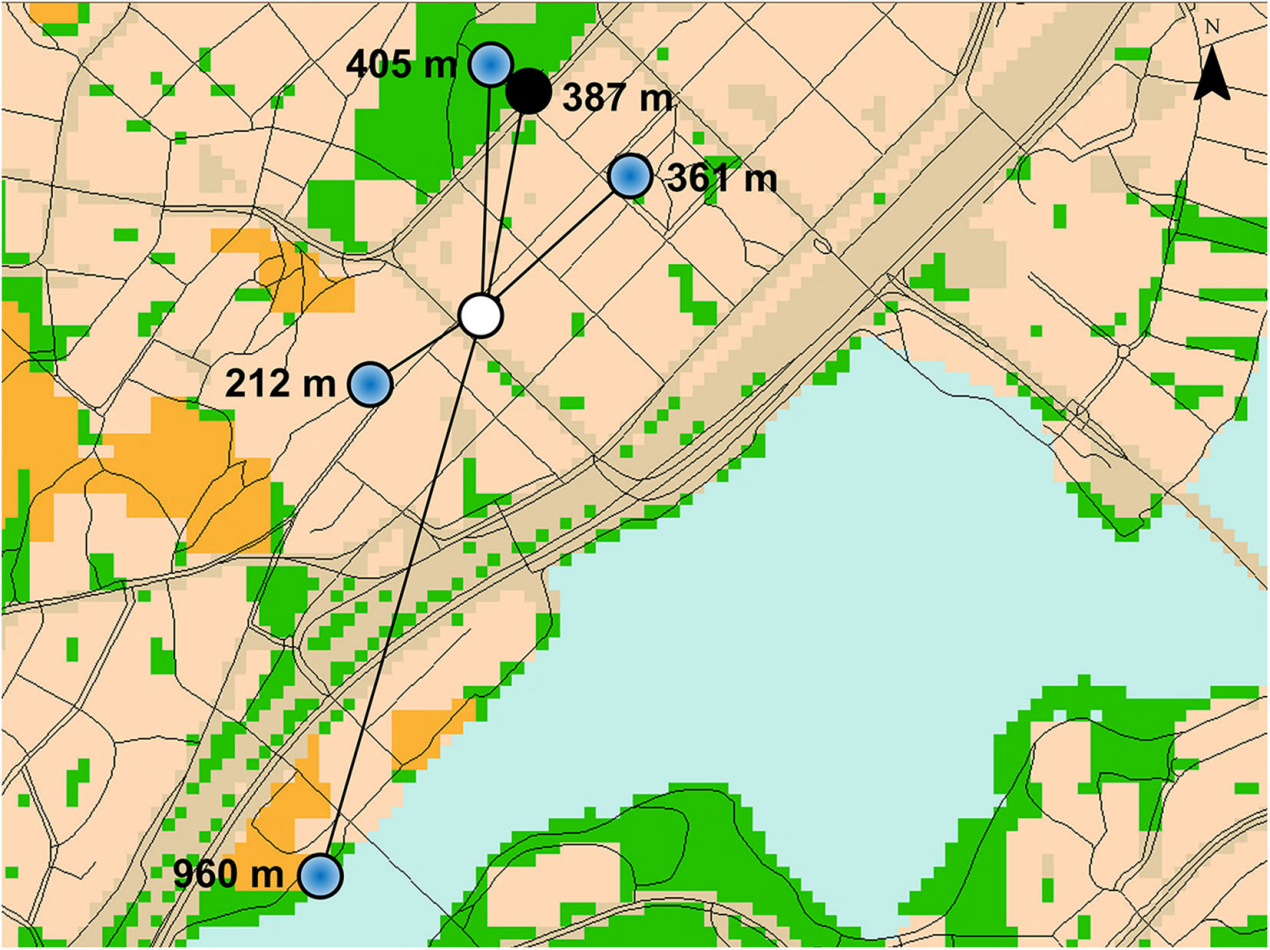
Visualization of the Euclidean distance from the home (white open circle) of a fictive participant to reported destinations (blue dot) and barriers (black dot) for outdoor mobility located on a map [geographical datasets used (30, 32)].

The PENFOM and PENBOM checklists were used to collect participant perceptions of environmental destinations and barriers to outdoor mobility in the neighborhood, respectively (13). For each item, participants were asked to indicate whether they perceived that the respective feature facilitated or hindered their outdoor mobility (yes vs. no). If an item was reported, the participant was subsequently asked to locate it on an online interactive map using the Maptionnaire tool (Mapita, Espoo, Finland). Considering the prevalence of computer illiteracy in this age group, an interviewer assisted participants technically with orientation on the map and navigation to desired locations. For this study, we selected from the PENFOM questionnaire 5 items considered as *neighborhood destinations*; that is, park or other green space, walking trail or skiing track, nature or lakeside, appealing scenery, and services such as shops, markets, or events nearby. From the PENBOM questionnaire, all locatable *neighborhood barriers* to outdoor mobility, 14 items in total, were used for the analyses; that is, poor street conditions, high curbs, lack of sidewalks, hills in nearby environment, lack of benches, poor lighting, noisy environment, busy traffic, dangerous cross-roads, vehicles on walkways, cyclists on walkways, insecurity caused by other pedestrians, snow and ice, and lack of benches in winter. Participants were allowed to provide more than one location for each item. For each participant, we computed Euclidean distances from home to all reported locations (visualized in Figure 1; expressed in units of 100 m) and used the distance to the most distantly located neighborhood destination and barrier, respectively, for further analyses (*maximal distance*). Furthermore, overall presence of destinations or barriers was determined (none reported vs. reported), thus, also including reporting destinations and barriers with unknown location owing to technical problems or participants' inability to locate features.

### Covariates

Participants' *age* and *sex* were derived from the population register. The number of self-reported physician-diagnosed *chronic conditions* was computed based on a 22-item checklist and an additional open question (33). The number of chronic conditions is a commonly used and recommended indicator of total disease burden and recommended when information on severity of diseases is lacking (34). *Physical performance* was measured using Short Physical Performance Battery (SPPB), comprising a balance, a 2.44-m walking, and a 5-time sit-to-stand test (33, 35). Each test was scored from zero to four using age- and sex-specific cut-off points and a sum score was computed (range 0–12). For five participants, the test was not conducted (e.g., because of wheelchair use or temporary restriction), and for one participant, one missing subscore for reasons unrelated to mobility was replaced by average score of the two remaining tests. *Cognitive function* was assessed using the Mini-Mental State Examination (MMSE) (36). For four participants, missing item scores for reasons unrelated to cognition were imputed using the average of available items. The MMSE score ranges from 0 to 30, and higher scores indicate better performance. In addition, *social support* was assessed using self-report questions of having a friend with whom to walk or run errands (yes vs. no) and living arrangement (lives alone vs. lives together with spouse, relative, or others).

### Statistical Analyses

Descriptive characteristics were compared between those categorized as having higher vs. lower levels of physical activity using Mann–Whitney *U*-tests or χ^2^-tests depending on the variable distribution. Characteristics are reported as medians and interquartile ranges or percentages.

Logistic regression analyses were used to test associations between physical activity and neighborhood destinations, and associations between physical activity and neighborhood barriers to outdoor mobility. First, the analyses were run including the variable overall presence, and then maximal distance from home was added to the model. All analyses were adjusted for age and sex (method enter), and subsequently, using forward conditional selection, adjusted for statistically significant covariates number of chronic conditions, SPPB score, MMSE score, and two dichotomous variables of social support. SPPB score was the sole covariate that statistically significantly contributed to all models and was thus reported as part of the final models.

In addition, we conducted the logistic regression models described before with categorized variables as independent variables. For this, we categorized participants based on presence and maximal distance to reported neighborhood destinations or barriers, respectively, as follows for 250, 500, 750, and 1,000 m buffer distances from home: (1) none reported (reference category); (2) reported, all within the respective buffer distance; (3) reported, at least one beyond the respective buffer distance; and (4) reported, but location unknown. Finally, we conducted sensitivity analyses comparing those reporting destinations or barriers beyond each buffer distance with those reporting them within the respective distance, i.e., category 3 vs. 2, thus excluding category 1 and 4 from the analyses.

Because of low numbers of reported barriers especially, it was not possible to study potential interaction effects between the neighborhood destinations and barriers to outdoor mobility. However, we did add to models of neighborhood destinations the variable indicating overall presence of perceived barriers (yes vs. no), but this did not markedly change the results (thus, not reported).

SPPS version 24 (IBM SPPS Statistics version 24, Armonk, NY, USA) was used for all statistical analyses and *p* < 0.050 was considered statistically significant.

## Results

Of the 206 participants in the MIIA study, 196 participants completed the map-based questionnaire on neighborhood destinations and barriers to outdoor mobility, four participants had ended the interview before the assessment, one was unable to respond, and in five cases technical problems related to the PC or server prevented data collection. Physical activity was assessed successfully in 194 participants, which left 185 participants with data on both the neighborhood environment and physical activity for the current analyses. Those who dropped out from the analyses did not differ from participants analyzed for any of the descriptive variables (data not shown).

Table 1 shows characteristics of participants. Participants who were more physically active were on average younger, they had lower SPPB scores and lower MMSE scores, and they were less frequently living alone.

**Table 1:** Descriptive characteristics of participants with lower and higher physical activity levels.

	**Lower** **physical activity (*n* = 103)**	**Higher** **physical activity (*n* = 82)**	***P*-value**
	**Median (IQR)**	**Median (IQR)**	
Age (years)	85.3 (8.3)	81.4 (5.5)	<0.001^a^
Chronic conditions (*n*)	4.0 (3.0)	4.0 (4.0)	Unable to compute^a^
SPPB score (p)	8.0 (4.0)	11.0 (1.0)	<0.001^a^
MMSE score (p)	26.0 (4.0)	27.5 (3.3)	0.002^a^
Sex (female, %)	62.1	50.0	0.132^b^
Friend for walking (yes, %)	50.5	59.8	0.208^b^
Living alone (yes, %)	68.0	48.8	0.013^b^

*IQR, interquartile range; SPPB, Short Physical Performance Battery*.

a *Mann–Whitney U-test*.

b *χ^2^-test*.

### Reporting of Neighborhood Destinations and Barriers to Outdoor Mobility

About 80% of participants reported at least one neighborhood destination for outdoor mobility, and for 93% of these reported destinations, a location was reported (Table 2). Neighborhood destinations were located at a median distance of 492 m from home (range 25 m−6.10 km), and the distance was longer for those reporting higher (median = 704 m, IQR = 634 m) than lower (median = 349 m, IQR = 458 m; *p* < 0.001) physical activity. Participants with higher physical activity more frequently reported neighborhood destinations in general (89%) and especially at longer distances from home (e.g., ≥500 m 55%) than those with lower activity (70 and 22%, respectively).

**Table 2:** Neighborhood destinations and barriers to outdoor mobility reported and their distance to home.

	**Distance**	**All (*****n*** **= 185)**		**Lower** **physical activity (*****n*** **= 103)**		**Higher** **physical activity (*****n*** **= 82)**		***P*-value**
	**From home**	***N***	**%**	***N***	**%**	***N***	**%**	
**Destinations**								<0.001
None reported		40	21.6	31	30.1	9	11.0	
Reported	<250 m	39	21.1	27	26.2	12	14.6	
	250–499 m	29	15.7	17	16.5	12	14.6	
	500–749 m	28	15.1	13	12.6	15	18.3	
	750–999 m	14	7.6	2	1.9	12	14.6	
	≥1,000 m	26	14.1	8	7.8	18	22.0	
	Location unknown	9	4.9	5	4.9	4	4.9	
**Barriers**								<0.001
None reported		82	44.3	35	34.0	47	57.3	
Reported	<250 m	54	29.2	38	36.9	16	19.5	
	250–499 m	19	10.3	14	13.6	5	6.1	
	500–749 m	8	4.3	4	3.9	4	4.9	
	750–999 m	2	1.1	1	1.0	1	1.2	
	≥1,000 m	3	1.6	0	0.0	3	3.7	
	Location unknown	17	9.2	11	10.7	6	7.3	

About half of participants reported at least one neighborhood barrier to outdoor mobility, and for 84% of these reported barriers, a location was reported (Table 2). Barriers were located at a median distance of 166 m from home (range 5 m−2.7 km), and the distance was similar regardless of physical activity (higher median = 193 m, IQR = 509 m; vs. lower median = 155 m, IQR = 218 m; *p* = 0.684). Participants with lower physical activity more frequently reported barriers to outdoor mobility in general (66%) and especially within 250 m from home (37%) than those with higher activity (43 and 20%, respectively).

### Logistic Regression Analyses—Neighborhood Destinations for Outdoor Mobility

Table 3 shows that only when not accounting for distance, reporting any neighborhood destination perceived to facilitate one's outdoor mobility increased the odds for higher physical activity [OR 3.22, 95% confidence interval (CI) 1.33–7.76] compared with reporting no destinations at all. Adjusting for SPPB score attenuated the association so that it was no longer statistically significant. Among those reporting neighborhood destinations, each 100-m distance between home and the most distant neighborhood destination increased the odds for higher physical activity by at least 11%, also when adjusted for SPPB score.

**Table 3:** Logistic regression models of overall presence and maximal distance from home to neighborhood destinations and barriers to outdoor mobility and odds ratios (OR) for higher physical activity (*n* = 185).

	**Overall presence**				**Presence and maximal distance**			
	**Age and sex adjusted**		**Fully adjusted**		**Age and sex adjusted**		**Fully adjusted**	
	**OR**	**95% CI**	**OR**	**95% CI**	**OR**	**95% CI**	**OR**	**95% CI**
**Destinations**								
None reported	1.00		1.00		1.00		1.00	
Reported	**3.22**	**1.33–7.76**	1.64	0.62–4.32	1.52	0.55–4.19	0.83	0.27–2.49
Maximal distance	–	–	–	–	**1.12**	**1.04–1.22**	**1.11**	**1.02–1.21**
Age	0.81	0.75–0.89	0.84	0.76–0.93	0.81	0.74–0.89	0.84	0.76–0.93
Sex	1.06	0.55–2.05	1.75	0.83–3.72	1.13	0.56–2.28	1.70	0.78–3.71
SPPB score	–	–	1.72	1.38–2.15	–	–	1.66	1.31–2.09
**Barriers**								
None reported	1.00		1.00		1.00		1.00	
Reported	**0.36**	**0.19–0.69**	**0.40**	**0.19–0.85**	**0.23**	**0.10–0.53**	**0.22**	**0.09–0.55**
Maximal distance	–	–	–	–	**1.16**	**1.01–1.32**	**1.21**	**1.04–1.40**
Age	0.81	0.74–0.89	0.84	0.76–0.93	0.80	0.73–0.88	0.84	0.76–0.93
Sex	1.12	0.58–2.18	1.63	0.76–3.49	1.36	0.66–2.78	1.80	0.80–4.07
SPPB score	–	–	1.73	1.39–2.16	–	–	1.72	1.36–2.19

*Statistically significant associations of main variables are bolded (p < 0.050)*.

*SPPB, Short Physical Performance Battery; 95% CI, 95% confidence interval*.

Table 4 shows that those reporting at least one neighborhood destination beyond 250 m had four times and those reporting at least one neighborhood destination beyond 500 m had six times the odds for higher physical activity than those reporting no destinations at all in the age- and sex adjusted model. Adjusting for SPPB score attenuated the associations to three times the odds for the 500-m distance, and it was no longer statistically significant for the 250-m distance. Reporting all destinations within 250 or 500 m from home was not associated with physical activity when compared with reporting no destinations. Sensitivity analyses showed that compared with those reporting all destinations within 500 m, those reporting at least one destination further away more than tripled the odds to report higher physical activity (age- and sex-adjusted model OR 3.98, 95% CI 1.15–6.81; fully adjusted model OR 3.73, 95% CI 1.56–8.93).

**Table 4:** Logistic regression models of neighborhood destinations and barriers to outdoor mobility categorized by distance from home and odds ratios (OR) for higher physical activity (*n* = 185).

		**Age and sex adjusted**		**Fully adjusted**	
		**OR**	**95% CI**	**OR**	**95% CI**
Destinations 250 m	None reported	1.00		1.00	
	All within	1.51	0.50–4.57	0.90	0.26–3.13
	≥1 beyond	**4.22**	**1.69–10.50**	1.95	0.71–5.33
	Location unknown	2.25	0.43–11.62	1.77	0.32–9.91
	Age	0.82	0.75–0.89	0.84	0.76–0.93
	Sex	1.16	0.59–2.31	1.78	0.83–3.81
	SPPB score	–	–	1.70	1.36–2.14
Destinations 500 m	None reported	1.00		1.00	
	All within	1.61	0.60–4.31	0.78	0.26–2.35
	≥1 beyond	**6.19**	**2.34–16.41**	**2.95**	**1.02–8.54**
	Location unknown	2.17	0.41–11.37	1.70	0.30–9.61
	Age	0.81	0.74–0.89	0.83	0.75–0.92
	Sex	1.27	0.63–2.55	2.01	0.92–4.41
	SPPB score	–	–	1.73	1.37–2.18
Destinations 750 m	None reported	1.00		1.00	
	All within	2.03	0.80–5.15	1.07	0.38–2.97
	≥1 beyond	**11.21**	**3.60–34.97**	**4.99**	**1.44–17.26**
	Location unknown	2.32	0.44–12.27	1.82	0.32–10.28
	Age	0.79	0.72–0.87	0.82	0.74–0.91
	Sex	1.08	0.54–2.18	1.62	0.75–3.54
	SPPB score	–	–	1.69	1.34–2.12
Destinations 1 km	None reported	1.00		1.00	
	All within	2.77	1.12–6.82	1.41	0.52–3.82
	≥1 beyond	**6.94**	**2.09–23.09**	3.07	0.82–11.49
	Location unknown	2.35	0.45–12.16	1.83	0.33–10.22
	Age	0.81	0.74–0.89	0.84	0.76–0.93
	Sex	1.05	0.53–2.05	1.67	0.78–3.58
	SPPB score	–	–	1.72	1.37–2.15
Barriers 250 m	None reported	1.00		1.00	
	All within	**0.31**	**0.14–0.69**	**0.30**	**0.12–0.74**
	≥1 beyond	0.48	0.19–1.19	0.56	0.20–1.55
	Location unknown	0.33	0.10–1.04	0.51	0.14–1.79
	Age	0.81	0.74–0.89	0.84	0.76–0.93
	Sex	1.14	0.58–2.25	1.65	0.76–3.59
	SPPB score	–	–	1.75	1.40–2.18
Barriers 500 m	None reported	1.00		1.00	
	All within	**0.30**	**0.14–0.62**	**0.31**	**0.14–0.72**
	≥1 beyond	0.96	0.26–3.45	0.96	0.23–3.99
	Location unknown	0.33	0.10–1.04	0.51	0.14–1.79
	Age	0.81	0.74–0.89	0.84	0.76–0.93
	Sex	1.14	0.58–2.5	1.61	0.74–3.49
	SPPB score	–	–	1.74	1.39–2.18

*Barrier reporting beyond 750 or 1,000 m was too rare to compute valid regression models. Statistically significant associations of main variables are bolded (p < 0.050)*.

*SPPB, Short Physical Performance Battery; 95% CI, 95% confidence interval*.

Those reporting neighborhood destinations beyond 750 or 1,000 m had increased odds for higher physical activity (OR 6–11), but confidence intervals were wide and group sizes relatively small.

### Logistic Regression Analyses—Neighborhood Barriers to Outdoor Mobility

Compared with reporting none, reporting any neighborhood barrier to outdoor mobility (regardless of distance) was associated with lower odds to report higher physical activity (OR 0.36, 95% CI 0.19–0.69; Table 3). Among those reporting barriers, each 100-m distance between the home and the most distant mobility barrier increased the odds to report higher physical activity by at least 16%.

Table 4 shows that those reporting neighborhood barriers within 250 or 500 m from home only (OR 0.31, 95% CI 0.14–0.69 and OR 0.30, 95% CI 0.14–0.62, respectively), but not those reporting barriers also further away, had markedly lower odds for higher physical activity than those reporting no neighborhood barriers at all. Further adjustment of the models for SPPB score did not markedly change the described associations. Sensitivity analyses showed that those reporting at least one barrier at or beyond 500 m from home tended to report higher physical activity than those reporting barriers solely within 500 m, although statistical significance was not reached (age- and sex-adjusted OR 3.23, 95% CI 0.91–11.44, and fully adjusted OR 3.03, 95% CI 0.79–11.62, respectively).

Because of a small number of participants reporting neighborhood barriers to outdoor mobility beyond 750 and 1,000 m (2.7%), it was not meaningful to conduct regression analyses using these buffer sizes.

## Discussion

A common assumption in research of older adults' physical activity behavior is that they move close to the home and, thus, that the environment close to home may motivate or hinder older adults' mobility and physical activity (12, 16, 20). This study using an interactive map-based questionnaire provides new information about spatial relations between neighborhood destinations and barriers to outdoor mobility relative to older adults' homes. The distance from home to neighborhood destinations facilitating outdoor mobility, rather than their presence *per se*, was associated with physical activity. Neighborhood destinations facilitating outdoor mobility, such as nature, parks, and services, were associated with higher physical activity especially when located further away from home, i.e., beyond 500 m. In contrast, outdoor mobility barriers, such as street quality and difficult terrain, were associated with markedly lower levels of physical activity, especially when located close to the home, i.e., within 250 or 500 m.

In line with previous research (25), distances of 500 m from home may not be sufficient to capture all destinations for outdoor mobility of an older person. The current study showed that reporting locations beyond 500 m from home especially correlated with higher physical activity. Correspondingly, research has shown that older adults moving further away from home generally are more physically active (37). A previous study based on traditional questionnaire data showed that reporting destinations within 10- to 20-min walk distance from home in a lower density city was associated with higher physical activity in adults aged 65 and over, thus suggesting that destinations are optimally located when within easy reach, but not too close to home (38). Although walking speeds vary in old age (39), it is likely that destinations located beyond 500 m from home, as reported in the current study, are situated within a 10- to 20-min walk time frame. However, other studies have shown that shorter distances to destinations may be beneficial on the long term, as reporting utilitarian destinations within 10 min from home was associated with better maintenance of walking for transportation 4 years later in adults 50–64 years old (18). In addition, objectively assessed proximity to services based on home and service locations was also associated with better maintenance of walking activity 3 years later in adults 67–84 years old (23). Differences between cross-sectional and longitudinal findings warrant further study.

In contrast to neighborhood destinations, neighborhood barriers to outdoor mobility were more commonly identified at relatively close distance from home. In line with previous research (7, 8), the current study shows that those reporting at least one barrier in the neighborhood more likely reported lower physical activity. When further looking at locations of reported barriers, we found that this association was true only when barriers were located within 250 or 500 m from home and not when barriers were located further away from home. Moreover, as a continuous variable, longer distances from home to reported barriers increased the odds for higher physical activity. This finding may be explained in several ways. Older adults with a restricted life space are known to report outdoor mobility barriers in the neighborhood more frequently than those moving further away from home (13). Outdoor mobility barriers located closer to home, including those related to poor walking conditions, may be related to avoidance of activities for example as a result of fear of falls (40). In the current study, only those moving further away from the home—and consequently more physically active (37)—were likely reporting barriers located further away from home. To perceive an outdoor mobility barrier, it needs to be relevant to one's outdoor mobility and one needs to be aware of it (41), thus, located in the area used by an individual. Moreover, barriers located further away from the home may be less limiting for physical activity and more easy to avoid, i.e., by taking alternative routes than those located closer to home. Taking a closer look at specific barriers revealed that especially lack of benches was reported at longer distances from home. Possibly, this suggests that older adults may need environmental support when moving further away from home. Considering that barriers are typically perceived only when the demands of the environment challenge the capacities of an individual (6), it is possible that any barrier reporting, including perceived barriers at long distances from home, point to early declines in functioning. It is unclear whether reporting perceived barriers located further away from home may potentially lead to avoidance of activity or whether individuals are able to modify their behaviors to overcome the challenge and maintain their activity regardless (42, 43).

The sample of the current study was on average well-over 80 years, an age where physical and cognitive limitations typically manifest. Previous studies have shown that associations between environmental features and physical activity differ for those with and without limitations in walking or physical function (29, 44). Adjusting the current analyses for an early indicator of functional decline, i.e., the SPPB score, clearly attenuated associations between neighborhood destinations and physical activity. Thus, in line with previous studies (24, 29), it seems that those with better function more frequently report neighborhood destinations and higher physical activity. Yet, the association found between neighborhood barriers to outdoor mobility and physical activity was virtually unaffected by adjustment for SPPB score. This contradicts the assumptions of the socioecological model (6), where declines in physical capacity are expected to increase the vulnerability to environmental demands, and thus, as a logical consequence, would affect barrier reporting. However, considering the fact that barrier perception also depends on use and awareness of the environment (41), it is possible that those with poorer function, and thus, less physically active, may not report neighborhood barriers to outdoor mobility as a result of infrequent moving through the neighborhood.

Cognitive function, chronic conditions, and social support were not associated with physical activity in any of the current regression models, and they did not affect associations between neighborhood destinations and barriers for outdoor mobility and physical activity. Partly the lack of associations may be related to the use of rather crude measures in the current study. Executive function, one domain of cognitive function involved in task planning and coordination, may be more proximal to motoric tasks and physical activity than general cognitive function, such as assessed with the MMSE (45). Furthermore, one single chronic condition with large debilitating effects on mobility, e.g., painful musculoskeletal or neurological conditions, may be more meaningful than overall chronic diseases burden (46). However, considering the difficulty to assess disease severity and impact in large epidemiological studies, general indicators of chronic disease burden are frequently used and recommended (34). In addition to the physical environment, aspects of the social environment may play an important role in physical activity (19). Social activities, such as visiting friends, may provide a reason to go out and having a companion to walk with may make it more enjoyable to leave home, and, as a consequence, facilitate physically active lifestyle (7, 37, 47). In the current study, participants with lower physical activity more frequently lived alone, and also, they were older and more often female, possibly related to widowhood. However, the other indicator of social support in the current study, that is, having a friend to walk or run errands with, did not differ according to physical activity level. Furthermore, based on these two indicators, social support was not associated with physical activity. Yet considering loneliness being a common problem in aging populations, relations of the social and physical environment and physical activity warrant further study.

Until recently, relations between perceived distance or proximity to services and physical activity were mainly based on questionnaire data without reference to the actual environment (20). Technological innovations led to the development of map-based questionnaires, but spatial measures of the perceived environment have rarely been used in research (48) and, to our knowledge, previously only in adults up to the age of 75 years (49). Our participants were markedly older (79–94 years) and were able to determine locations for most of the features hindering and facilitating their outdoor mobility on a map when provided with technical assistance by an interviewer. Independent completion of map-based questionnaires will be possible in the near future, as younger generations are more familiar with the use of digital devices. Map-based questionnaires seem a feasible alternative for collecting place-based data from participants, as GPS data collection currently burdens both researchers (data cleaning and analyses) and participants (e.g., continuous charging of device) (50). GPS and map-based self-report data do not fully coincide, but still provide reasonable estimates of distances traveled by older adults (50, 51).

Strengths of the current study are the population-based sample of adults above 75 years. There were relatively few missing data and characteristics of participants and non-respondents were studied and did not markedly differ. We used a novel method to collect data from older adults using map-based questionnaires and thereby provide new insights in spatial relations in physical activity research.

Limitations of the study were the rather limited sample size, which did not enable us to look at subgroups of age, sex, or function or to thoroughly adjust for potential confounders. This study comprised a culturally relatively homogenous sample; therefore, generalizability of these results to cultural settings beyond Finland needs to be established. Only self-reported measures for the neighborhood environment and for physical activity were available for analyses. Physical activity derived from self-report questionnaires is typically overestimated, but the use of accelerometers poses higher commitment of study participants and staff, and its accurateness may be challenged by slow movement patterns typical for older adults (52). The measure of physical activity was non-specific, covering both utilitarian and recreational walking as well as other physical activities, but different environmental features may be associated with such types of physical activity (16, 53). Perceived neighborhood features may not accurately reflect the actual environment (22, 29), but may be more proximal to physical activity behavior of the individual than more objectively assessed measures of the environment (21). Ideally, studies should include both objective and perceived measures of the environment and physical activity to provide a comprehensive picture. The current study does not account for residential self-selection, which may theoretically bias the results (54). However, with half of participants already living in the same home for 23 years and with marked urbanization of the study area in the past decennia, choices made at the time of moving to the current home may no longer be relevant. Distances from home to neighborhood destinations and barriers were measured over a straight line (Euclidean distance), thus likely underestimating actual distances along the road network, which are more complicated to compute (55). Regardless, distances of 250 and 500 m from home, as used in the current study, are likely within walkable distance along the road network as well. Yet it is possible that distances are perceived differently by each person depending on function and habits, thus associations with perceived distances require further study.

## Conclusions

The current study shows that Finnish older adults move at distances beyond 500 m from home, and that those reporting neighborhood destinations and barriers to outdoor mobility beyond such distances are likely more physically active. Outdoor mobility barriers seem to limit physical activity only when located closer to the home, that is, within 250 or 500 m from home. Based on the current study, collecting spatial data using map-based questionnaires seems feasible even in older populations. Utilizing such data expands the possibilities for scientific research on person–environment interactions and may help to inform urban planning about designing environments conducive of active aging. Comprehensive measures including perceptions and objective measures of environmental features and distances are needed to capture the full picture of spatial relations and person–environment interactions in physical activity relative to older adults' homes. Future research should broaden the scope to also cover activities beyond physical activity, i.e., investigating active aging more in general and in more diverse settings.

## Data Availability Statement

Due to ethical restrictions pseudonymized datasets are available only upon request from Professor Taina Rantanen (taina.rantanen@jyu.fi). External collaborators may use data upon agreement on the terms of data use and publication of results.

## Ethics Statement

The studies involving human participants were reviewed and approved by Ethical Committee of the University of Jyväskylä. The patients/participants provided their written informed consent to participate in this study.

## Author Contributions

EP, JE, and TR: acquisition of data. KK: spatial analyses. EP: statistical analyses and drafting the manuscript. KK, JE, MS, MR, and TR: critically revising the manuscript and substantially contributing to its contents. All authors: concept and design of study and interpretation of results.

## Conflict of Interest

The authors declare that the research was conducted in the absence of any commercial or financial relationships that could be construed as a potential conflict of interest.

## References

[1] World Health Organization Active Ageing. A Policy Framework. Geneva. Geneva: World Health Organization (2002) 12040973

[2] EU European Innovation Partnership on Active and Healthy Ageing. Available online at: https://ec.europa.eu/eip/ageing/home_en (accessed February 06, 2018).

[3] Piercy KL Troiano RP Ballard RM Carlson SA Fulton JE Galuska DA. The Physical activity guidelines for Americans. JAMA. (2018) 320:2020–8. 10.1001/jama.2018.14854 30418471 PMC9582631

[4] Rantanen T Portegijs E Kokko K Rantakokko M Tormakangas T Saajanaho M. Developing an assessment method of active aging: university of Jyvaskyla active aging scale. J Aging Health. (2019) 31:1002–24. 10.1177/0898264317750449 29291660 PMC6572587

[5] Fishman E Bocker L Helbich M. Adult active transport in the Netherlands: an analysis of its contribution to physical activity requirements. PLoS ONE. (2015) 10:e0121871. 10.1371/journal.pone.0121871 25849902 PMC4388541

[6] Lawton MP Nahemow L. Ecology and aging process. In: Eisdorfer C Lawton MP editors. The Psychology of Adult Development and Aging. Washington DC: American Psychology Association (1973). p. 619–74. 10.1037/10044-020

[7] Tsai LT Rantakokko M Portegijs E Viljanen A Saajanaho M Eronen J. Environmental mobility barriers and walking for errands among older people who live alone vs. with others. BMC Public Health. (2013) 13:1054. 10.1186/1471-2458-13-1054 24207063 PMC4226209

[8] Portegijs E Rantakokko M Viljanen A Rantanen T Iwarsson S. Perceived and objective entrance-related environmental barriers and daily out-of-home mobility in community-dwelling older people. Arch Gerontol Geriatr. (2017) 69:69–76. 10.1016/j.archger.2016.11.011 27889590

[9] WHO Global Network of Age-Friendly Cities and Communities. Available online at: http://www.who.int/ageing/age_friendly_cities_network/en/ (accessed January 07, 2015).

[10] Global Age-Friendly Cities: A Guide. France: World Health Organization (2007).

[11] Buffel T Phillipson C. A manifesto for the age-friendly movement: developing a new urban agenda. J Aging Soc Policy. (2018) 30:173–92. 10.1080/08959420.2018.1430414 29364777

[12] Moran M Van Cauwenberg J Hercky-Linnewiel R Cerin E Deforche B Plaut P. Understanding the relationships between the physical environment and physical activity in older adults: a systematic review of qualitative studies. Int J Behav Nutr Phys Act. (2014) 11:79. 10.1186/1479-5868-11-79 25034246 PMC4119420

[13] Rantakokko M Iwarsson S Portegijs E Viljanen A Rantanen T. Associations between environmental characteristics and life-space mobility in community-dwelling older people. J Aging Health. (2015) 27:606–21. 10.1177/0898264314555328 25326130

[14] Winters M Voss C Ashe MC Gutteridge K McKay H Sims-Gould J. Where do they go and how do they get there? Older adults' travel behaviour in a highly walkable environment. Soc Sci Med. (2015) 133:304–12. 10.1016/j.socscimed.2014.07.006 25017579

[15] Davis MG Fox KR Hillsdon M Coulson JC Sharp DJ Stathi A. Getting out and about in older adults: the nature of daily trips and their association with objectively assessed physical activity. Int J Behav Nutr Phys Act. (2011) 8:116. 10.1186/1479-5868-8-116 22018626 PMC3213209

[16] Barnett DW Barnett A Nathan A Van Cauwenberg J Cerin E Council on Environment and Physical Activity (CEPA) - Older Adults working group. Built environmental correlates of older adults' total physical activity and walking: a systematic review and meta-analysis. Int J Behav Nutr Phys Act. (2017) 14:103 10.1186/s12966-017-0558-z 28784183 PMC5547528

[17] Eronen J von Bonsdorff M Rantakokko M Rantanen T. Environmental facilitators for outdoor walking and development of walking difficulty in community-dwelling older adults. Eur J Aging. (2014) 11:67–75. 10.1007/s10433-013-0283-7 28804315 PMC5549184

[18] Sugiyama T Cerin E Mridha M Koohsari MJ Owen N. Prospective associations of local destinations and routes with middle-to-older aged adults' walking. Gerontologist. (2018) 58:121–9. 10.1093/geront/gnx088 28575195

[19] Kestens Y Wasfi R Naud A Chaix B. “Contextualizing Context”: reconciling environmental exposures, social networks, and location preferences in health research. Curr Environ Health Rep. (2017) 4:51–60. 10.1007/s40572-017-0121-8 28188604

[20] Sugiyama T Neuhaus M Cole R Giles-Corti B Owen N. Destination and route attributes associated with adults' walking: a review. Med Sci Sports Exerc. (2012) 44:1275–86. 10.1249/MSS.0b013e318247d286 22217568

[21] Weden MM Carpiano RM Robert SA. Subjective and objective neighborhood characteristics and adult health. Soc Sci Med. (2008) 66:1256–70. 10.1016/j.socscimed.2007.11.041 18248865

[22] Gebel K Bauman A Owen N. Correlates of non-concordance between perceived and objective measures of walkability. Ann Behav Med. (2009) 37:228–38. 10.1007/s12160-009-9098-3 19396503

[23] Gauvin L Richard L Kestens Y Shatenstein B Daniel M Moore SD. Living in a well-serviced urban area is associated with maintenance of frequent walking among seniors in the VoisiNuAge study. J Gerontol B Psychol Sci Soc Sci. (2012) 67:76–88. 10.1093/geronb/gbr134 22227735

[24] Rantakokko M Iwarsson S Manty M Leinonen R Rantanen T. Perceived barriers in the outdoor environment and development of walking difficulties in older people. Age Ageing. (2012) 41:118–21. 10.1093/ageing/afr136 22086965

[25] Prins RG Pierik F Etman A Sterkenburg RP Kamphuis CB van Lenthe FJ. How many walking and cycling trips made by elderly are beyond commonly used buffer sizes: results from a GPS Study. Health Place. (2014) 27C:127–33. 10.1016/j.healthplace.2014.01.012 24603010

[26] Hinrichs T Keskinen KE Pavelka B Eronen J Schmidt-Trucksass A Rantanen T. Perception of parks and trails as mobility facilitators and transportation walking in older adults: a study using digital geographical maps. Aging Clin Exp Res. (2019) 31:673–83. 10.1007/s40520-018-01115-0 30666515

[27] Siltanen S Rantanen T Portegijs E Tourunen A Poranen-Clark T Eronen J. Association of tenacious goal pursuit and flexible goal adjustment with out-of-home mobility among community-dwelling older people. Aging Clin Exp Res. (2019) 31:1249–56. 10.1007/s40520-018-1074-y 30449015 PMC6682663

[28] Rantanen T Portegijs E Viljanen A Eronen J Saajanaho M Tsai LT. Individual and environmental factors underlying life space of older people - study protocol and design of a cohort study on life-space mobility in old age (LISPE). BMC Public Health. (2012) 12:1018. 10.1186/1471-2458-12-1018 23170987 PMC3534010

[29] Portegijs E Keskinen KE Tsai LT Rantanen T Rantakokko M. Physical limitations, walkability, perceived environmental facilitators and physical activity of older adults in Finland. Int J Environ Res Public Health. (2017) 14:333. 10.3390/ijerph14030333 28327543 PMC5369168

[30] Finnish Transport Agency Digiroad Road and Street Database, R-Format 2013. Helsinki: CSC—IT Center for Science Ltd., & Finnish Transport Agency (2013).

[31] Portegijs E Sipila S Viljanen A Rantakokko M Rantanen T. Validity of a single question to assess habitual physical activity of community-dwelling older people. Scand J Med Sci Sports. (2017) 27:1423–30. 10.1111/sms.12782 27747944

[32] Finnish Environment Institute SYKE (partly Metla, Mavi, LIVI, VRK, MML Topographic Database (2012 May)). Corine Land Cover (CLC) 2012 and Finnish National Dataset (20 m). Helsinki: CSC—IT Center for Science Ltd., & Finnish Environment Institute SYKE (2012).

[33] Portegijs E Rantakokko M Mikkola TM Viljanen A Rantanen T. Association between physical performance and sense of autonomy in outdoor activities and life-space mobility in community-dwelling older people. J Am Geriatr Soc. (2014) 62:615–21. 10.1111/jgs.12763 24655124

[34] Nosikov A Gudex G editors. EUROHIS: Developing Common Instruments for Health Surveys. Amsterdam: WHO Regional Office for Europe; IOS Press (2003).

[35] Guralnik JM Simonsick EM Ferrucci L Glynn RJ Berkman LF Blazer DG. A short physical performance battery assessing lower extremity function: association with self-reported disability and prediction of mortality and nursing home admission. J Gerontol. (1994) 49:M85–94. 10.1093/geronj/49.2.M85 8126356

[36] Folstein MF Folstein SE McHugh PR. “Mini-mental state.” A practical method for grading the cognitive state of patients for the clinician. J Psychiatr Res. (1975) 12:189–98. 10.1016/0022-3956(75)90026-6 1202204

[37] Portegijs E Tsai LT Rantanen T Rantakokko M. Moving through greater life-space areas and objectively measured physical activity of older people. PLoS ONE. (2015) 10:e0135308. 10.1371/journal.pone.0135308 26252537 PMC4529301

[38] Boakye-Dankwa E Barnett A Pachana NA Turrell G Cerin E. Associations between latent classes of perceived neighborhood destination accessibility and walking behaviors in older adults of a low-density and a high-density city. J Aging Phys Act. (2019) 27:553–64. 10.1123/japa.2018-0297 30676201

[39] Bohannon RW Williams Andrews A. Normal walking speed: a descriptive meta-analysis. Physiotherapy. (2011) 97:182–9. 10.1016/j.physio.2010.12.004 21820535

[40] Lee S Lee C Ory MG Won J Towne SD Jr Wang S. Fear of outdoor falling among community-dwelling middle-aged and older adults: the role of neighborhood environments. Gerontologist. (2018) 58:1065–74. 10.1093/geront/gnx123 28958081

[41] Portegijs E Rantakokko M Edgren J Salpakoski A Heinonen A Arkela M. Effects of a rehabilitation program on perceived environmental barriers in older patients recovering from hip fracture: a randomized controlled trial. Biomed Res Int. (2013) 2013:769645. 10.1155/2013/769645 23986910 PMC3748419

[42] Rantakokko M Portegijs E Viljanen A Iwarsson S Rantanen T. Mobility modification alleviates environmental influence on incident mobility difficulty among community-dwelling older people: a two-year follow-up study. PLoS ONE. (2016) 11:e0154396. 10.1371/journal.pone.0154396 27104750 PMC4841513

[43] Baltes PB Baltes MM. Psychological perspectives on successful aging: the model of selective optimization with compensation. In: Baltes PB Baltes MM editors. Successful Aging: Perspectives From the Behavioral Sciences. Cambridge: Cambridge University Press (1990) p. 1–34. 10.1017/CBO9780511665684.003

[44] Keskinen KE Rantakokko M Suomi K Rantanen T Portegijs E. Nature as a facilitator for physical activity: defining relationships between the objective and perceived environment and physical activity among community-dwelling older people. Health Place. (2018) 49:111–9. 10.1016/j.healthplace.2017.12.003 29306140

[45] Eggermont LH Milberg WP Lipsitz LA Scherder EJ Leveille SG. Physical activity and executive function in aging: the MOBILIZE Boston study. J Am Geriatr Soc. (2009) 57:1750–6. 10.1111/j.1532-5415.2009.02441.x 19702618 PMC3286835

[46] Vancampfort D Koyanagi A Ward PB Rosenbaum S Schuch FB Mugisha J. Chronic physical conditions, multimorbidity and physical activity across 46 low- and middle-income countries. Int J Behav Nutr Phys Act. (2017) 14:6. 10.1186/s12966-017-0463-5 28100238 PMC5241915

[47] Tsai LT Rantakokko M Viljanen A Saajanaho M Eronen J Rantanen T. Associations between reasons to go outdoors and objectively-measured walking activity in various life-space areas among older people. J Aging Phys Act. (2016) 24:85–91. 10.1123/japa.2014-0292 25951008

[48] Perchoux C Chaix B Cummins S Kestens Y. Conceptualization and measurement of environmental exposure in epidemiology: accounting for activity space related to daily mobility. Health Place. (2013) 21:86–93. 10.1016/j.healthplace.2013.01.005 23454664

[49] Laatikainen TE Hasanzadeh K Kytta M. Capturing exposure in environmental health research: challenges and opportunities of different activity space models. Int J Health Geogr. (2018) 17:29. 10.1186/s12942-018-0149-5 30055616 PMC6064075

[50] Schmidt T Kerr J Kestens Y Schipperijn J. Challenges in using wearable GPS devices in low-income older adults: can map-based interviews help with assessments of mobility? Transl Behav Med. (2019) 9:99–109. 10.1093/tbm/iby009 29554353

[51] Hinrichs T Zanda A Fillekes MP Bereuter P Portegijs E Rantanen T (in review). Map-based assessment of older adults' life- space: validity and reliability (2020). 10.1186/s11556-020-00253-7 PMC7700712 33292160

[52] Schrack JA Cooper R Koster A Shiroma EJ Murabito JM Rejeski WJ. Assessing daily physical activity in older adults: unraveling the complexity of monitors, measures, and methods. J Gerontol A Biol Sci Med Sci. (2016) 71:1039–48. 10.1093/gerona/glw026 26957472 PMC4945889

[53] Cerin E Nathan A van Cauwenberg J Barnett DW Barnett A Council on Environment and Physical Activity (CEPA) - Older Adults working group. The neighbourhood physical environment and active travel in older adults: a systematic review and meta-analysis. Int J Behav Nutr Phys Act. (2017) 14:15 10.1186/s12966-017-0471-5 28166790 PMC5294838

[54] Cao X Residential self-selection in the relationships between the built environment and travel behavior: introduction to the special issue. J Transport Land Use. (2014) 7:1–3. 10.5198/jtlu.v7i3.726

[55] Shahid R Bertazzon S Knudtson ML Ghali WA. Comparison of distance measures in spatial analytical modeling for health service planning. BMC Health Serv Res. (2009) 9:200. 10.1186/1472-6963-9-200 19895692 PMC2781002

